# Nutritional strategy underlying plant specialization to gypsum soils

**DOI:** 10.1093/aobpla/plad041

**Published:** 2023-06-29

**Authors:** Andreu Cera, Gabriel Montserrat-Martí, Sara Palacio

**Affiliations:** Centre for Applied Ecology ‘Prof. Baeta Neves’ (CEABN-InBIO), School of Agriculture, University of Lisbon, Tapada da Ajuda, Lisbon, 1349-017, Portugal; Departamento Biodiversidad y Restauración, Instituto Pirenaico de Ecología, Consejo Superior de Investigaciones Científicas, Avenida Nuestra Señora de la Victoria 16, Jaca, 22700, Spain; Departamento Biodiversidad y Restauración, Instituto Pirenaico de Ecología, Consejo Superior de Investigaciones Científicas, Avenida de Montañana 1005, Zaragoza, 50059, Spain; Departamento Biodiversidad y Restauración, Instituto Pirenaico de Ecología, Consejo Superior de Investigaciones Científicas, Avenida Nuestra Señora de la Victoria 16, Jaca, 22700, Spain

**Keywords:** Arid environments, extreme soils, mineral nutrition, plant–soil interactions, semi-arid environments, soil specialization

## Abstract

Gypsum soils are amongst the most widespread extreme substrates of the world, occurring in 112 countries. This type of hypercalcic substrate has a suite of extreme physical and chemical properties that make it stressful for plant establishment and growth. Extreme chemical properties include low plant-available nitrogen and phosphorus and high plant-available sulphur and calcium, which impose strong nutritional imbalances on plants. In spite of these edaphic barriers, gypsum soils harbour rich endemic floras that have evolved independently on five continents, with highly specialized species. Plants that only grow on gypsum are considered soil specialists, and they have a foliar elemental composition similar to the elemental availability of gypsum soils, with high calcium, sulphur and magnesium accumulation. However, the physiological and ecological role of the unique foliar elemental composition of gypsum specialists remains poorly understood, and it is unknown whether it provides an ecological advantage over other generalist species on gypsum soils. This article reviews available literature on the impact of gypsum soil features on plant life and the mechanisms underlying plant adaptation to gypsum environments. We conclude with a hypothesis on the potential role of the nutritional strategy underlying plant specialization to gypsum soils: Gypsum specialists primarily use SO_4_^2–^ as a counter anion to tolerate high Ca^2+^ concentrations in cells and avoid phosphorus depletion, which is one of the most limiting nutrients in gypsum soils.

## Teaser

Gypsum soils are unique substrates with nutritional imbalances, which occur in drylands worldwide. Nutritional imbalances limit plant life, but also favour plant diversification. Gypsum soils harbour rich endemic floras with highly specialized species. Gypsum specialists accumulate calcium, sulphur and magnesium in leaves, which are highly available in gypsum soils. However, it is still unknown why gypsum specialists have such foliar accumulation and whether it provides an ecological advantage. This article reviews the available literature on plant adaptation to gypsum soils, and concludes with a hypothesis on the role of this accumulation: gypsum specialists use sulphur to tolerate high calcium concentrations, and thus improve phosphorus nutrition, which is the most limited nutrient in gypsum soils.

## Introduction

Extreme soils often have mineral nutrient imbalances compared to plant nutritional requirements. To overcome imbalances of nutrients in excess, plants mainly display different physiological tolerance mechanisms. As described in saline, calcicole and metalliferous soils (Munns and Tester 2008; Tran *et al.* 2020; Lux *et al.* 2021), plants can: (i) exclude nutrients at the root level; (ii) sequester and accumulate excess nutrients in below-ground organs, thereby restricting translocation to above-ground plant parts; or (iii) translocate excess elements to the shoots, which requires tolerating high concentrations of these elements in the above-ground plant parts.

These physiological mechanisms convey tolerance to edaphic endemic plant species that specialize in growing on chemically extreme soil. Chemically extreme soil specialists generally tolerate excess elements in above-ground organs, while their soil generalist sister species generally exclude nutrient uptake at the root level, or sequester them in below-ground organs. However, there are multiple examples of soil specialists and generalists with one or multiple physiological tolerance mechanisms (Kazakou *et al.* 2008). For example, saline soils contain an excess of plant-available sodium (Na) and chlorine (Cl). Many halophytes, as saline specialist species, are foliar Na and Cl accumulators, but glycophytes, as generalists, are non-foliar accumulators in saline soils (Munns and Tester 2008; Matinzadeh *et al.* 2019). The Na and Cl accumulation by halophytes is a physiological tolerance mechanism for osmotic adjustment (Flowers *et al.* 1977; Munns and Tester 2008). Similarly, calcareous soils display an excess of plant-available calcium (Ca) in soils. Calcicole species, as specialists, tolerate Ca excess in leaves (White and Broadley 2003), while calcifuge species may exclude Ca at the root level or sequester it in roots, to prevent translocation of excess Ca to leaves (Kotula *et al.* 2021). The different behaviour between calcicoles and calcifuges has consequences on phosphorus (P) nutrition (Lux *et al.* 2021). When calcifuge species are forced to grow in calcareous soils, they have an excessive Ca uptake, leading to the precipitation of calcium phosphate in their tissues, which produces P shortage (Hayes *et al.* 2019). Contrastingly, calcicole species have various physiological mechanisms to reduce Ca activity in cells, such as compartmentalization, or complexing the Ca with organic acids as oxalates, malates or flavonoids (Kinzel 1989; White and Broadley 2003) to avoid precipitation of calcium phosphate and safeguard P nutrition (Hayes *et al.* 2019).

Gypsum (CaSO_4_·2H_2_O) soils are amongst the most widespread extreme substrates in the world, and also present nutritional stresses for plants due to their unique soil composition (Casby-Horton *et al.* 2015). Despite these edaphic barriers, gypsum soils support diverse plant communities, with species distributed across several taxonomic orders and families (Moore *et al.* 2014). Many of these species have a high affinity for gypsum soils (Mota *et al.* 2011; Pérez-García *et al.* 2017; Musarella *et al.* 2018; Ochoterena *et al.* 2020), known as gypsophiles (Mota *et al.* 2016), and are considered as soil specialists (Palacio *et al.* 2007). Nevertheless, the flora of gypsum environments is not only made up by gypsum specialists, it also includes species with wide ecological amplitudes in terms of soil affinity (Meyer 1980), known as gypsovags (Mota *et al.* 2016). Some gypsophiles have gypsovags as sister taxa in several lineages (Moore *et al.* 2014). Both groups of species share functional traits, and tend to be species with predominantly stress-tolerant adaptations (Hodgson *et al.* 1994) due to the harshness of gypsum environments. However, gypsophiles differ from gypsovags by their foliar composition, accumulating elements found in excess in gypsum soils, as do other edaphic specialists with their sister generalist taxa. This foliar composition is shared by gypsophiles from different families and regions of the world (Duvigneaud and Denaeyer-De Smet 1966; Alvarado 1995; Palacio *et al.* 2007, 2022; Bolukbasi *et al.* 2016; Muller *et al.* 2017; Merlo *et al.* 2019). In contrast to the well-known physiological tolerance mechanism of other soil specialists, the physiological and ecological role of the foliar composition of gypsophiles remains elusive (Cera *et al.* 2021*b*). Additionally, it is unknown whether it provides an ecological advantage to specialist species over other generalist species on gypsum soils.

The aim of this paper is to review the available literature on the impact of gypsum soil features on plant life and the mechanisms underlying plant adaptation to gypsum environments. We focused on the physiological mechanisms of plants growing in gypsum soils as the main differential trait between gypsophiles and gypsovags. We further propose a hypothesis on the role of the nutritional strategy underlying plant specialization to gypsum soils based on the interaction between Ca excess and P nutrition, and also discuss other possible roles. We conclude with considerations for future studies to elucidate the underlying mechanisms of plant adaptation to gypsum environments, and to understand the differential soil affinity between gypsophiles and gypsovags.

## Gypsum Soil Features Impact on Plant Life

Gypsum soils are special substrates where gypsum mineral content is greater than 40 % (in dry weight), which impacts soil physicochemical properties (Herrero and Porta 2000). The moderate solubility of gypsum (about 2.4 g L^–1^) is an important property that modifies soil characteristics, resulting in highly dynamic soil environments, with dissolution–precipitation sequences that alter physicochemical properties (Casby-Horton *et al.* 2015). High gypsum contents generate abnormally high ionic concentrations of Ca^2+^ and SO_4_^2–^ in the soil solution. The high Ca^2+^ saturates the cation exchange complex, leading to nutrient immobilization and low availability (FAO 1990; Guerrero-Campo *et al.* 1999). Compared to other alkaline soils with high Ca content, gypsum soils have higher Ca cation activity than soils with calcite (CaCO_3_) as the main mineral component due to the higher solubility of gypsum (Harmsen 1984). The result is higher Ca availability and lower P availability to plants due to precipitation of P as Ca_3_(PO_4_)_2_ (calcium phosphate) in soils (Novozamsky and Beek 1976), which is added to the lower concentration of inorganic P in gypsum soils as compared to soils with calcite (Cera *et al.* 2021*a*, *b*). Gypsum soils only occur in drylands in arid, temperate and continental climates (Eswaran and Zi-Tong 1991; Herrero *et al.* 2009; Casby-Horton *et al.* 2015), where low precipitation prevents gypsum from being leached from the soil, affecting plant performance and distribution (Moore *et al.* 2014; Escudero *et al.* 2015).

The effects of gypsum soils on plants have been previously explored by several authors (i.e. Hernando Fernández *et al.* 1963; Meyer 1986; Verheye and Boyadgiev 1997). Gypsum soils impose severe nutrient limitations for plant life (Hernando Fernández *et al.* 1963), although the chemical properties of gypsum soils do not produce osmotic stress in plants (Casby-Horton *et al.* 2015). The most distinctive feature of gypsum soils is the high concentration of Ca^2+^ and SO_4_^2–^ ions in the soil solution. This results in a decrease in nutrient availability and subsequently reduced plant potassium (K), P and iron uptake (FAO 1990), and increases the foliar concentration of Ca and sulphur (S) of most plants (Boukhris and Lossaint 1975; Palacio *et al.* 2007; Salmerón-Sánchez *et al.* 2014). Similarly, a recent common garden experiment demonstrated that plants growing in gypsum soils displayed higher leaf S and magnesium (Mg) and lower P and K concentrations than those growing on calcareous soils (Cera *et al.* 2021*b*). These studies demonstrated that gypsum remarkably alters plant nutrition and ultimately limits plant growth (Cera *et al.* 2021*b*). Therefore, species growing in gypsum soils have developed physiological tolerance strategies to cope with the excess of Ca, S and Mg and low availability of N, K and P in the soil (Duvigneaud and Denaeyer-De Smet 1966).

## The Nutritional Strategy of Gypsophiles

Gypsophiles generally display a unique foliar composition with a remarkable accumulation of excess nutrients from gypsum soils in above-ground organs (Palacio *et al.* 2022). Accordingly, gypsophiles display higher foliar S and Mg (and marginally higher Ca) and lower K concentrations than gypsovags (Palacio *et al.* 2007, 2022). The increased accumulation of S and Mg of gypsophiles also occurs in stems and coarse roots, although both groups show similar concentrations in fine roots (Cera *et al.* 2022*a*). Gypsophiles are hence leaf accumulators, whereas generalist gypsovag species seem to exclude S, Mg and Ca uptake at the fine root level, as an avoidance strategy to gypsum.

As Ca and S accumulators (Merlo *et al.* 2019), gypsophiles primarily store S as sulphate in leaves (Cera *et al.* 2022*a*). Sulphate appears to be stored in the vacuole (Abdallah *et al.* 2010) in a saturated solution with Ca, and not in calcium sulphate crystals, as described in other papers (i.e. Palacio *et al.* 2014; He *et al.* 2015). Plants would actively prevent calcium sulphate crystallization by regulating water content and compartmentalizing Ca accumulation. Many gypsophiles have succulent leaves (Moore *et al.* 2014), and gypsophiles are also foliar Mg accumulators (Merlo *et al.* 2019; Palacio *et al.* 2022). Even at low concentrations, Mg^2+^ is a strong inhibitor of the nucleation and growth kinetics of calcium sulphate crystals (Rabizadeh *et al.* 2017). Therefore, the physiological role of this strategy of gypsophiles could be related to Ca sequestration, using sulphate as a chelating compound, and Mg as an inhibitor of calcium sulphate crystallization to increase solubility in cells (A. Cera *et al.*, unpubl. data).

## Gypsophiles Use Sulphates to Manage Ca Accumulation and Safeguard P Nutrition

We hypothesize that gypsophiles use sulphate as a counter anion to assimilate high Ca concentrations in cells and manage Ca accumulation and P depletion. Hence, plant specialization to gypsum soils would be related to plant specialization to alkaline soils with high Ca and low P, since gypsum soils are hypercalcic with remarkably limited P availability (Cera *et al.* 2021*a*). Similarly, calcicolous species display several physiological solutions to tolerate high Ca in cells and avoid interactions with P nutrition, as organic acid metabolism (White and Broadley 2003). We suggest that gypsophiles use sulphate as a chelating compound, as described in the literature for non-gypsophile species (Kinzel 1989; He *et al.* 2015; Robson *et al.* 2017). This does not preclude gypsophiles from using other physiological mechanisms to tolerate high Ca in cells. For example, some gypsophiles belonging to Caryophyllaceae display oxalate metabolism (Palacio *et al.* 2014; Muller *et al.* 2017), and most of them have likely evolved from calcicolous lineages (Heiden *et al.* 2022; Palacio *et al.* 2022).

To understand plant specialization to gypsum soils, it is necessary to compare gypsophiles with their generalist sister taxa in their ability to grow on gypsum soils. Most gypsum generalist species are calcicoles (Bolukbasi *et al.* 2016; Muller *et al.* 2017; Palacio *et al.* 2022). Thus, these species have pre-adaptive physiological mechanisms to tolerate excess Ca in their cells (White and Broadley 2003). However, they produced less biomass when growing on gypsum than on calcareous soil (without gypsum) in a common garden experiment, while gypsophiles produced similar biomass (Cera *et al.* 2022*a*). Hence, it seems that adaptations to establish and survive on limestone and marble soils should convey preadaptation to establish, survive and complete the life cycle on gypsum soils (Cera *et al.* 2021*b*), but not to achieve optimal performance. Furthermore, in the Chihuahuan Desert and to a lesser extent in the Mediterranean Basin, gypsophiles tend to accumulate higher Ca in leaves than gypsovags when growing on similar gypsum soils (Muller *et al.* 2017; Palacio *et al.* 2022).

Here we suggest that the nutritional strategy of gypsophiles, including increased Ca, Mg and S accumulation, should affect their P nutrition. Gypsophiles may accumulate more Ca in leaves than gypsovags, thus avoiding precipitation of Ca phosphate. Although there are few studies of plant-available P in gypsum soils, there are some recent studies that support this idea. Gypsophiles have less arbuscular mycorrhizal colonization than gypsovags (Palacio *et al.* 2012; Cera *et al.* 2021*a*), and consequently have a lower reliance on the strategy of mycorrhizae for P acquisition, which is very costly in carbon. Furthermore, in contrast to gypsophiles, when gypsovags are grown in gypsum soil, they display lower P concentration than when they are grown in calcareous soil (Cera *et al.* 2021*b*). Thus, gypsophiles are physiologically adapted to gypsum soil, which is a hypercalcic soil with harsher conditions than other calcareous soils. These specialists may have developed a physiological tolerance mechanism to regulate Ca accumulation and P restriction typical of calcareous soils, and accentuated in gypsum soils. Such physiological tolerance mechanism may involve using excess S and Mg to regulate excess Ca and prevent its precipitation with P. This tolerance mechanism should lead to a better performance of gypsophiles than gypsovags in gypsum soils, but will need to be tested in further studies (see below).

## Other Physiological Implications for S Accumulation by Gypsophiles

Sulphur accumulation by gypsophiles may have other physiological implications rather than improved Ca tolerance and enhanced P nutrition. On the one hand, it has been described that the chemical conditions of gypsum soils do not produce osmotic stress in plants (Casby-Horton *et al.* 2015), compared to saline soils, and gypsophiles do not show higher water-use efficiencies than gypsovags (Sánchez-Martín *et al.* 2021). However, many times gypsum soils appear mixed with saline soils, for example in Australia, Iran, the Chihuahuan Desert or Morocco (Pérez-García *et al.* 2017; Taleb and Fennane 2019). In those cases, there are species with high affinity for these soils, referred to as halogypsophiles (Ochoterena *et al.* 2020). These species are accumulators of Na, Ca and S, and remain little studied, but they may assimilate sulphate with Ca and Na, as plants in other systems such as saline soils (Reich *et al.* 2017, 2018). Such accumulation may have a physiological role in osmotic adjustments, because sulphate accumulation has been demonstrated to play a role in osmotic adjustments and drought in other ecosystems (Rivoal and Hanson 1994; Gallardo *et al.* 2014; Usmani *et al.* 2020). However, these ideas remain unexplored in gypsum systems.

Another unexplored adaptation of gypsophiles linked to foliar S accumulation is the possible role of S-rich chemical compounds to deter herbivores. Sulphur accumulation, either as sulphate or organic molecules like glucosinolates (Ernst 1990), may decrease palatability for herbivores (Palacio *et al.* 2014) by reducing the energetic and nutritional values of leaves. Plant–herbivore interactions can sometimes be central to edaphic specialization (Fine *et al.* 2004), as is likely the case for gypsum soils in some parts of the world (Cera *et al.* 2022*b*). The harsh soil features of gypsum are associated with disturbed habitats (Braun-Blanquet and Bolòs 1958; Guerrero-Campo *et al.* 1999), often produced by herbivores. Sheep disturbance favours the assemblage of gypsophiles over gypsovags in gypsum outcrops, and has been shown to promote foliar S accumulation in gypsum plant communities (Cera *et al.* 2022*c*), results that could be explained by a potential herbivore-deterrent role of S accumulation.

## Considerations for Future Studies

Advances in the physiological knowledge of gypsum plants, and particularly specialists, are relevant for the study of plants associated to special soils. Similarly, previous studies showed how plant species associated to serpentine or saline soils served as test models to understand the importance of plant–soil interactions in shaping the distribution, abundance and evolution of plants. In this review, we suggest several hypotheses on the physiological role of the elemental composition shared by gypsophiles, but further studies are needed to verify them. For example, it is crucial to assess the potential ecological costs and advantages of the foliar accumulation of Ca, S and Mg observed in gypsophiles. Describing the P nutrition strategies of plants growing in gypsum soils would be important to understand gypsum specialization, as few studies have paid attention to this key mineral nutrient in gypsum soils (Cera *et al.* 2021*a*). Then, it would be very important to relate the accumulation of Ca, sulphate and Mg to the P nutrition in plants growing in gypsum soils, as other studies did on calcifuge–calcicole species (Lee 1998; Zohlen and Tyler 2004; Hayes *et al.* 2019; Kotula *et al.* 2021). Further studies should also focus on sulphate accumulation in leaves as a physiological mechanism to promote Ca accumulation. It should be analysed in an evolutionary context, as a trait common to all gypsophilous lineages, but also in relation to the molecular basis of such accumulation, and the mechanisms underlying it. In addition, to elucidate the benefits of habitat specialization to gypsum, all these proposed studies should compare pairs of sister taxa with different affinity for gypsum soils and measure plant fitness (e.g. Sianta and Kay 2019).

The validation or refutation of the proposed hypotheses will help to make progress in the knowledge of gypsum plants. We believe ecophysiological knowledge of plant specialization to gypsum soils is needed to improve the conservation and restoration of gypsum ecosystems, favouring abiotic and biotic factors that promote the performance of gypsum plants. At the same time, such knowledge can aid in better understanding plant responses to factors other than gypsum soils, such as climate change, disturbances as fire or herbivory, or changes in land use. In addition, it can contribute to understand better the evolution of gypsum lineages, focusing on the traits that ultimately explain plant specialization to gypsum.

## Data Availability

No new data were generated for this research.

## Notes

**Plants, Ecosystems & Climate. Chief Editor:** Mary Heskel
